# Epizootic Hemorrhagic Disease in White-Tailed Deer, Canada

**DOI:** 10.3201/eid2504.180743

**Published:** 2019-04

**Authors:** Samantha E. Allen, Jamie L. Rothenburger, Claire M. Jardine, Aruna Ambagala, Kathleen Hooper-McGrevy, Nicole Colucci, Tara Furukawa-Stoffer, Stacey Vigil, Mark Ruder, Nicole M. Nemeth

**Affiliations:** University of Guelph and Canadian Wildlife Health Cooperative, Guelph, Ontario, Canada (S.E. Allen, J.L. Rothenburger, C.M. Jardine, N.M. Nemeth);; Canadian Food Inspection Agency, Winnipeg, Manitoba, Canada (A. Ambagala, K. Hooper-McGrevy);; Canadian Food Inspection Agency, Lethbridge, Alberta, Canada (N. Colucci, T. Furukawa-Stoffer);; University of Georgia, Athens, Georgia, USA (S. Vigil, M. Ruder, N.M. Nemeth)

**Keywords:** bluetongue, orbivirus, viruses, epizootic hemorrhagic disease, epizootic hemorrhagic disease virus, bluetongue virus, white-tailed deer, ruminants, Canada, Culicoides, RT-PCR, zoonosis, BTV, EHDV, vector-borne infections

## Abstract

Epizootic hemorrhagic disease affects wild and domestic ruminants and has recently spread northward within the United States. In September 2017, we detected epizootic hemorrhagic disease virus in wild white-tailed deer, *Odocoileus virginianus*, in east-central Canada. *Culicoides* spp. midges of the subgenus *Avaritia* were the most common potential vectors identified on site.

Epizootic hemorrhagic disease viruses (EHDVs) and bluetongue viruses (BTVs) are *Culicoides* spp. midge–transmitted orbiviruses that represent an imminent threat to the health of ruminant livestock and wildlife. For susceptible ruminants, EHDV and BTV infections can result in high rates of illness and death, leading to severe economic hardship to the agricultural sector (*1*). These viruses have a historical geographic range of 40°N–50°N and 35°S, following the distribution of the *Culicoides* vectors. However, the epidemiology of these pathogens is changing, with decades of northward expansion into areas of Europe and North America with immunologically naive hosts (*1**–**3*).

In Canada, EHDV has rarely and sporadically been detected in the southern portions of British Columbia, Alberta, and Saskatchewan (*4*). We report the detection of EHDV in white-tailed deer, *Odocoileus virginianus*, in east-central Canada, providing further evidence of the northern range expansion of orbiviruses within North America.

On September 7, 2017, two wild white-tailed deer carcasses were found in a seminatural area adjacent to a river and ravine in London, Ontario, Canada (42.9849°N, 81.2453°W). The carcasses were submitted for diagnostic investigation to the Ontario-Nunavut region of the Canadian Wildlife Health Cooperative. Both deer had gross lesions consistent with epizootic hemorrhagic disease, including multiorgan petechial and ecchymotic hemorrhages on serosal surfaces (*5*). Spleen, lung, and liver samples from both deer were submitted to the National Centre for Foreign Animal Diseases in Winnipeg. All samples were positive for EHDV genomes by reverse transcription PCR (RT-PCR) (*6*). The serotype of the virus was identified by serotype-specific conventional RT-PCR followed by Sanger sequencing. EHDV-2 was isolated from spleen samples of both deer. Two additional deer carcasses were identified in the area but were not submitted for diagnostic testing. Tissues from an additional 14 deer carcasses that originated in southern Ontario during September 23–November 28, 2017, showed negative results for EHDV by RT-PCR, as part of enhanced surveillance conducted by the Ontario Ministry of Natural Resources and Forestry and the Canadian Wildlife Health Cooperative.

In addition, we conducted serosurveillance across southern Ontario in the fall of 2017 with samples collected by private veterinarians. All live animal samples were collected by using protocols approved by the Animal Care Committee, University of Guelph (approval no. 3529). Cattle were from Chatham-Kent County, adjacent to Middlesex County, and were born and had lived their whole lives in Canada. Results revealed 15 cattle with antibodies to EHDV-2 by ELISA and serum neutralization tests (S.E. Allen, unpub. data). 

We also conducted a targeted survey for adult *Culicoides* midges in the area of the white-tailed deer deaths by using four 2770 UV LED CDC light traps (BioQuip Products, http://www.bioquip.com) for 16 trap-nights during September 9–13, 2017. We taxonomically identified 31 individual *Culicoides* spp. midges (*7*), including subgenus *Avaritia* (n = 22), *C. haematopotus* (n = 5), *C. stellifer* (n = 2), *C. venustus* (n = 1), and *C. crepuscularis* (n = 1) midges.

The northern expansion of EHDV and BTV in the midwestern and northeastern regions of the United States in recent years (*1**,**8*) has increased the likelihood of incursion into eastern Canada. The EHDV occurrence in white-tailed deer in Ontario we report supports the need to maintain vigilance. Although this initial occurrence was limited spatially and temporally, future cases may be more widespread in wildlife and livestock. Furthermore, this localized EHDV-2 occurrence was within 72 km of the US border of Michigan and coincided with concurrent and widespread EHDV-2 and EHDV-6 outbreaks across much of the eastern United States during July–October, 2017 (D.E. Stallknecht, University of Georgia, pers. comm, 2018 Jan 10).

EHDV may have been introduced to Ontario through the movement of infected vectors or ruminants, and its detection in Ontario may represent the northern edge of the EHDV outbreak in the eastern United States. Alternatively, low levels of previously undetected EHDV transmission may have been transient or ongoing across a wider region, including Ontario, for an unknown period. In either case, this event may preclude a much more widespread and higher impact EHDV outbreak, such as occurred with BTV in Europe and with EHDV in the United States (*2**,**8*).

We identified numerous *Culicoides* spp. midges at the site where the EHDV-infected white-tailed deer carcasses were recovered. However, *C. sonorensis* midges, the only confirmed EHDV vector species in North America (*1**,**9*), was not among these. This species was recently identified in Ontario (*10*), but the density and distribution of this species in the province, as well as its role in regional EHDV transmission, is currently unknown. *Culicoides* spp. composition within areas of past EHDV outbreaks varies widely; therefore, additional *Culicoides* spp. midges may serve as competent EHDV vectors. However, this possibility has not been confirmed (*1**,**9*). In addition to vector competence, the climatic limitations for the survival and successful breeding and establishment of current *Culicoides* spp. populations in the region remain to be investigated (*1**,**8*). These factors indicate the need for ongoing *Culicoides* spp. midge and deer mortality surveillance in the region.
